# Diphtheria Anti-Toxin Specific for Influenza

**Published:** 1918-11

**Authors:** 


### Diphtheria Anti-Toxin Specific for Influenza.

Dr. Louis J. Pint, former State bacteriologist and at present connected with the research laboratory of the University of Chicago, told the Chicago Medical Society he had succeeded in isolating the germs responsible for the so-called influenza epidemic, which is sweeping the country, and that the regulation diphtheria anti-toxin is an absolute specific for the disease.

He said the epidemic was mainly caused by the present war diet and especially by the curtailment of the usual consumption of sugar. Dr. Pint said he had treated seventy-five cases with the diphtheria anti-toxin without the loss of a single case.

After reading the report of Dr. Louis J. Pint's discovery, Dr. A. W. Carnes of Dallas declared that the announcement probably will have a great effect on the influenza situation.

"It is highly probable that the regulation diphtheria anti-toxin is a specific," said Dr. Carnes. "The anti-toxin has proven a specific for different diseases and I would not be at all surprised if the anti-toxin is an absolute cure and no doubt its use will be nation-wide immediately in an effort to check the spread of the influenza germ."

Dr. Carnes said he also believed the finding of Dr. Pint that the war diet and lack of sugar was partly the cause of the germ. He emphasized the point that Dr. Pint is a bacteriologist of note and that his discovery is of more than ordinary importance.

